# Supplementary material for: Multi-Region Microdialysis Imaging Platform Revealed Dorsal Raphe Nucleus Calcium Signaling and Serotonin Dynamics during Nociceptive Pain
Source: Int J Mol Sci. 2023 Apr 3;24(7):6654. doi: 10.3390/ijms24076654 (PMC10094999; doi:10.3390/ijms24076654)
Supplement: Supplementary file 1 [file ijms-24-06654-s001.zip › ijms-2286687-supplementary.pdf]

Supplementary

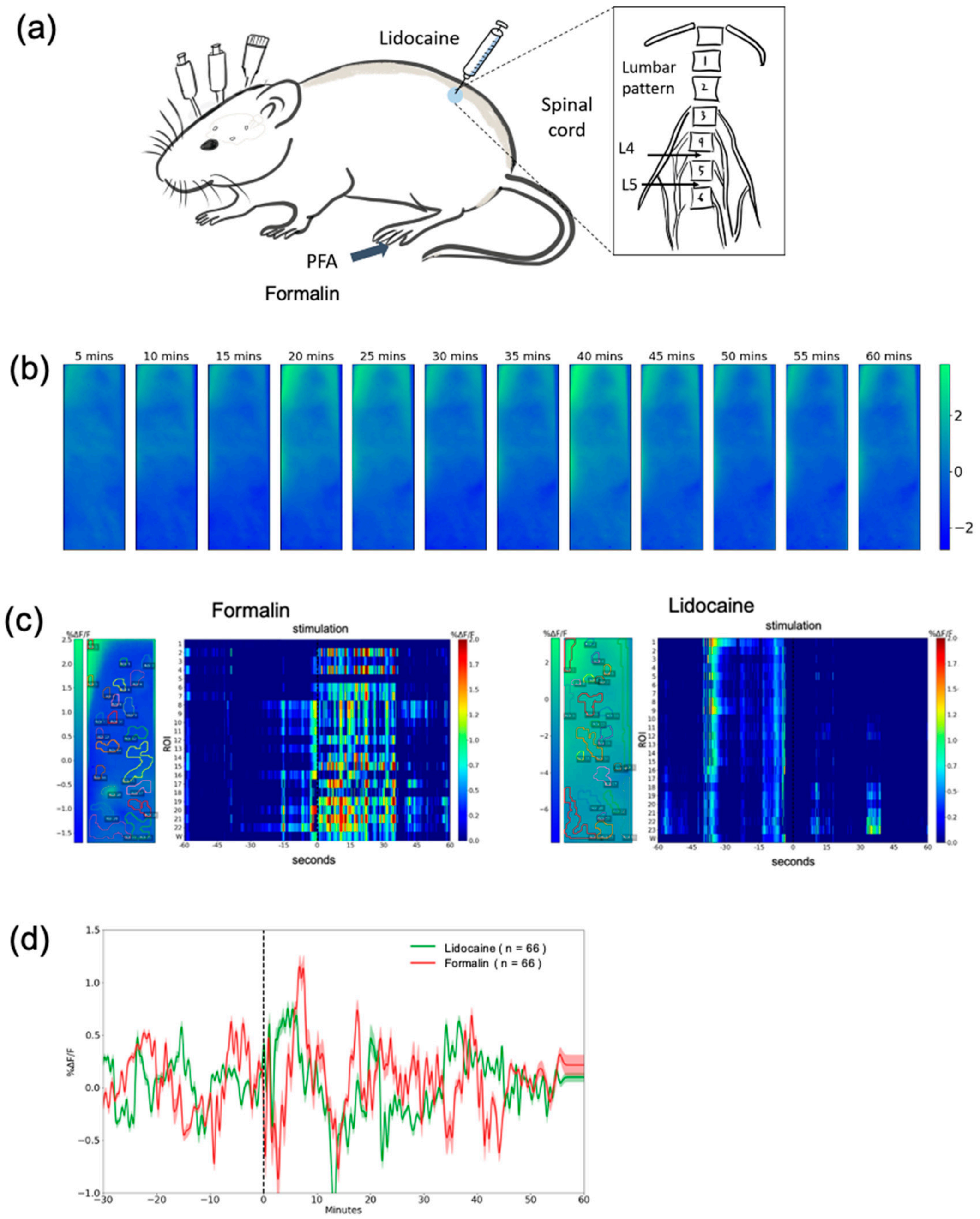

Supplementary Figure S1. Lidocaine injection into the spinal cord prior to formalin stimulation was able to suppress neural activity in the brain. (a) The schematic diagram showing the injection of lidocaine in the spinal cord between the groove of the L5 and L6 vertebrae, (b) Representative 5 minutes time-lapse of fluorescence changes after the stimulation in the Lidocaine group, (c) Representative heatmap of the Formalin and Lidocaine group, showing fluorescence changes ($\Delta F/F_0$) 60 seconds before and after the injection of formalin and lidocaine. This figure shows the

responsiveness of DRN immediately after formalin injection, (d) Average fluorescence changes of a genetically encoded calcium indicator (G-CaMP6) [27] after the intra-plantar injection of formalin and PBS (60 minutes). The dashed-black line shows the injection.

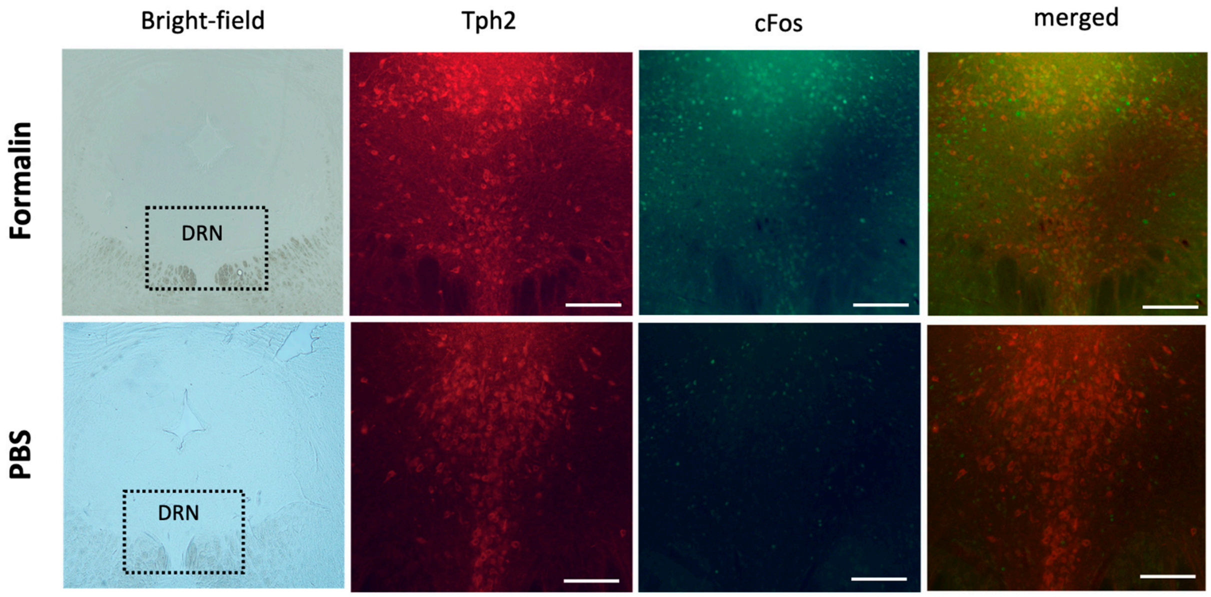

Supplementary Figure S2. Immunostaining results in the DRN. The dotted line square in the bright filed images shows the location of the DRN. The brain slices (40 µm) of DRN were stained with cFos and TPH2 to visualize activated neurons and serotonergic neurons, respectively. Scale bar: 200 µm.

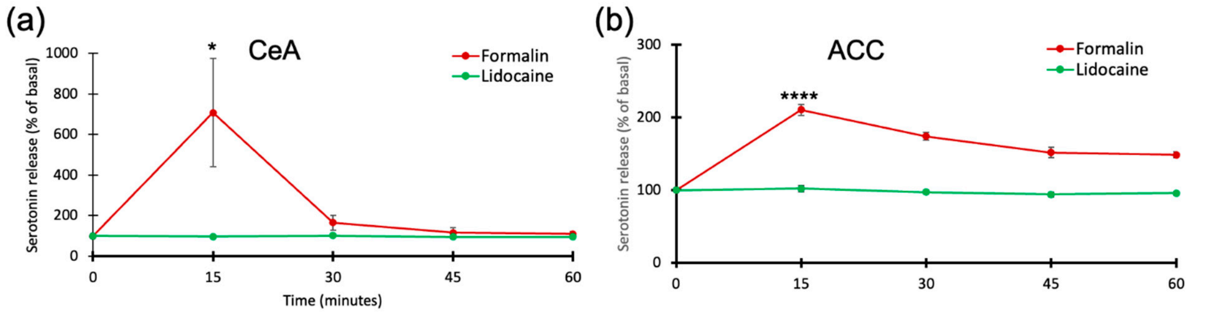

Supplementary Figure S3. Serotonin release comparing between formalin and lidocaine injection. (a) Serotonin release in the CeA, (b) Serotonin release in the ACC. Data are presented as mean ± SEM. Statistical analysis was conducted using unpaired t-test (n=5). *p<0.05, ****p<0.0001.

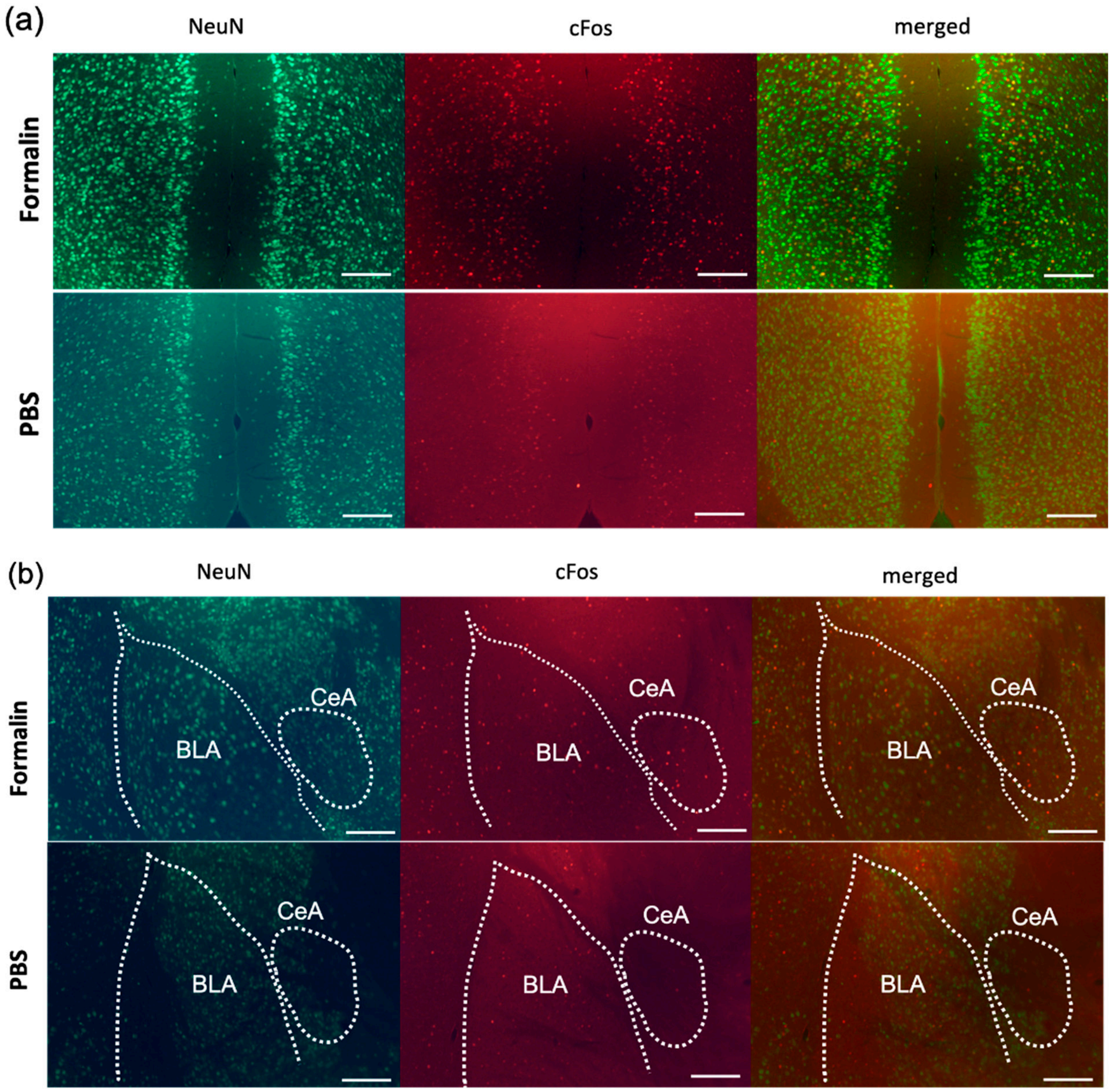

Supplementary Figure S4. Immunostaining results in the CeA and the ACC after formalin and PBS stimulation. (a) The brain slices (40 µm) of ACC were stained with NeuN and cFos antibodies for visualization of neuronal cell bodies and activated neurons. (b) CeA were stained with NeuN and cFos antibodies for visualization of neuronal cell bodies and activated neurons. Scale bar: 200 µm. CeA is the central nucleus of the amygdala, and BLA is the basolateral amygdala.

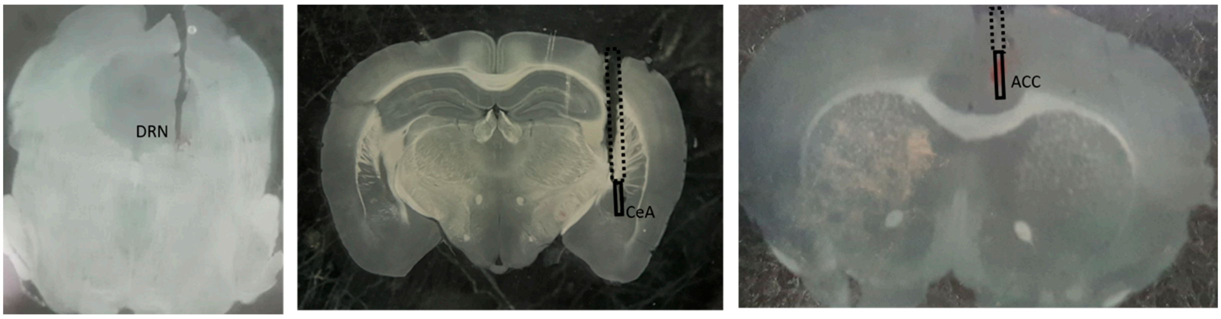

Supplementary Figure S5. Brain slicing for device confirmation, from left to right: device implantation in the DRN, the CeA, and the ACC, 40 μm thickness. The dashed line in the CeA and the ACC shows the probe, and the black line shows the membrane.
